# Drug cytotoxicity screening using human intestinal organoids propagated with extensive cost-reduction strategies

**DOI:** 10.1038/s41598-023-32438-2

**Published:** 2023-04-03

**Authors:** Yu Takahashi, Yu Inoue, Shintaro Sato, Takayoshi Okabe, Hirotatsu Kojima, Hiroshi Kiyono, Makoto Shimizu, Yoshio Yamauchi, Ryuichiro Sato

**Affiliations:** 1 Food Biochemistry Laboratory, Department of Applied Biological Chemistry, Graduate School of Agricultural and Life Sciences, The University of Tokyo, 1-1-1 Yayoi, Bunkyo-ku, Tokyo, 113-8657 Japan; 2 Department of Microbiology and Immunology, School of Pharmaceutical Sciences, Wakayama Medical University, Wakayama, 640-8156 Japan; 3 Department of Virology, Research Institute for Microbial Diseases, Osaka University, Osaka, 565-0871 Japan; 4 Drug Discovery Initiative, The University of Tokyo, Tokyo, 113-0033 Japan; 5 Mucosal Immunology and Allergy Therapeutics, Institute for Global Prominent Research, Future Medicine Education and Research Organization, Chiba University, Chiba, 263-8522 Japan; 6 Nutri-Life Science Laboratory, Department of Applied Biological Chemistry, Graduate School of Agricultural and Life Sciences, University of Tokyo, 1-1-1 Yayoi, Bunkyo-ku, Tokyo, 113-8657 Japan

**Keywords:** Biological techniques, Biotechnology, Cell biology, Drug discovery, Stem cells

## Abstract

Organoids are regarded as physiologically relevant cell models and useful for compound screening for drug development; however, their applications are currently limited because of the high cost of their culture. We previously succeeded in reducing the cost of human intestinal organoid culture using conditioned medium (CM) of L cells co-expressing Wnt3a, R-spondin1, and Noggin. Here, we further reduced the cost by replacing recombinant hepatocyte growth factor with CM. Moreover, we showed that embedding organoids in collagen gel, a more inexpensive matrix than Matrigel, maintains organoid proliferation and marker gene expression similarly when using Matrigel. The combination of these replacements also enabled the organoid-oriented monolayer cell culture. Furthermore, screening thousands of compounds using organoids expanded with the refined method identified several compounds with more selective cytotoxicity against organoid-derived cells than Caco-2 cells. The mechanism of action of one of these compounds, YC-1, was further elucidated. We showed that YC-1 induces apoptosis through the mitogen-activated protein kinase/extracellular signal-regulated kinase pathway, the mechanism of which was distinct from cell death caused by other hit compounds. Our cost-cutting methodology enables large-scale intestinal organoid culture and subsequent compound screening, which could expand the application of intestinal organoids in various research fields.

## Introduction

Phenotypic screening, which is based on cell phenotypes rather than molecular mechanisms, has contributed significantly to drug discovery^1^. One of the key characteristics of this assay is the adoption of physiologically relevant cell models that accurately reflect the in vivo biology^2,3^. Although established cell lines have been used in the research fields of molecular biology, pharmacology, and compound screening, they do not necessarily represent conserved signaling pathways in native cells or tissues. In contrast, native cells, such as stem cell-derived cells and primary cells, may serve as a better physiological system than the conventionally used cell lines. However, they have limitations in terms of culture scalability due to the loss of proliferative capacity, accessibility to human tissues, and reproducibility of differentiation and/or maturation states.

Organoids are defined as three-dimensional structures derived from (pluripotent) stem cells, progenitor cells, and/or differentiated cells that self-organize through cell–cell and cell–matrix interactions to mimic the architecture and function of native tissues in vitro^4^. They are expected to function as biological tools to replace animals and have industrial applications, such as regenerative medicine. Small intestinal organoids were the first established organoids with proliferative capacities in mice^5^ and humans^6,7^. Previously, we have succeeded in improving the differentiation efficiency of human induced pluripotent stem (iPS) cells into small intestinal organoids with ileum-like properties^8,9^. Additionally, we established monolayers of intestinal epithelial cells (IECs) from human iPS cell-derived intestinal organoids (hiPSOs)^10^ and primary human ileum organoids (hPIOs)^11^. Although organoids differentiated from iPS cells are reported to be functionally less mature than organoids from adult tissues^12^, we confirmed that several key functions specific to the intestinal tract, such as glucose transport, apolipoprotein B-48 secretion, and cytochrome P450 induction, which were not observed in colon adenocarcinoma-derived Caco-2 cells, were conserved in hiPSO-derived IECs^11^, suggesting their usefulness in screening studies on intestinal function and metabolism. Since organoid culture requires expensive recombinant proteins, we previously developed a single line of mouse L cells simultaneously overexpressing Wnt3a, R-spondin1, and Noggin (L-WRN cells) by lentiviral infection and successfully reduced the culture cost by using the culture supernatant (conditioned medium, CM) as the organoid growth medium^8^. However, further cost reduction is desired for assays that require large-scale cultures, such as screening studies.

Matrigel, a commercially available extracellular matrix (ECM) extracted from mouse sarcoma that contains many types of growth factors, has been routinely used for the culture of human organoids of the digestive system, including the stomach^13^, intestine^6^, liver^14^, and pancreas^15^. Despite its frequent use, it is accompanied by lot-to-lot variation and contains unknown humoral factors, which hinders human studies, including clinical trials^16^.

In this study, we succeeded in reducing the culture cost of human intestinal organoids and organoid-derived IECs using CM from mouse L cells exogenously expressing hepatocyte growth factor (HGF), together with Wnt3a, R-spondin1, and Noggin (L-WRNH cells). In addition, we replaced Matrigel with type I collagen gel as an ECM to embed organoids for further cost reduction. We conducted compound screening with intestinal organoids cultured using a combination of these methods and discovered several compounds with selective cytotoxicity against organoid-derived IECs. In this work, we have established an intestinal organoid culture at a greatly reduced cost, which enables drug screening and makes conducting other intestinal organoid applications more feasible, presumably leading to studies that are currently difficult to conduct owing to budget issues, especially in academia.

## Results

### Human intestinal organoids can be cultured with CM from L-WRNH cells

In a previous study, we successfully established L cells that stably overexpressed Wnt3a, R-spondin1, and Noggin at each required concentration^8^. The culture medium for human intestinal organoids was routinely prepared by adding 50 ng/mL human recombinant HGF to fourfold diluted CM harvested from L-WRN cells, which enabled a proliferation rate similar to that of organoids cultured with WRN recombinant proteins. Although HGF is seemingly not essential for human intestinal organoid culture^6,17^, we previously found that it significantly increased organoid growth, as observed in primary human colon organoids^17^, which would be beneficial for assays that require large numbers of organoids. Additionally, we showed that L-WRNH cells, whose culture supernatant contained approximately 200 ng/mL HGF, were established by infecting L-WRN cells with a human HGF lentivirus^8^. However, we did not examine whether CM prepared from L-WRNH cells could be applied to human intestinal organoid culture for further cost reduction by replacing recombinant HGF in the culture medium.

**Figure 1:**
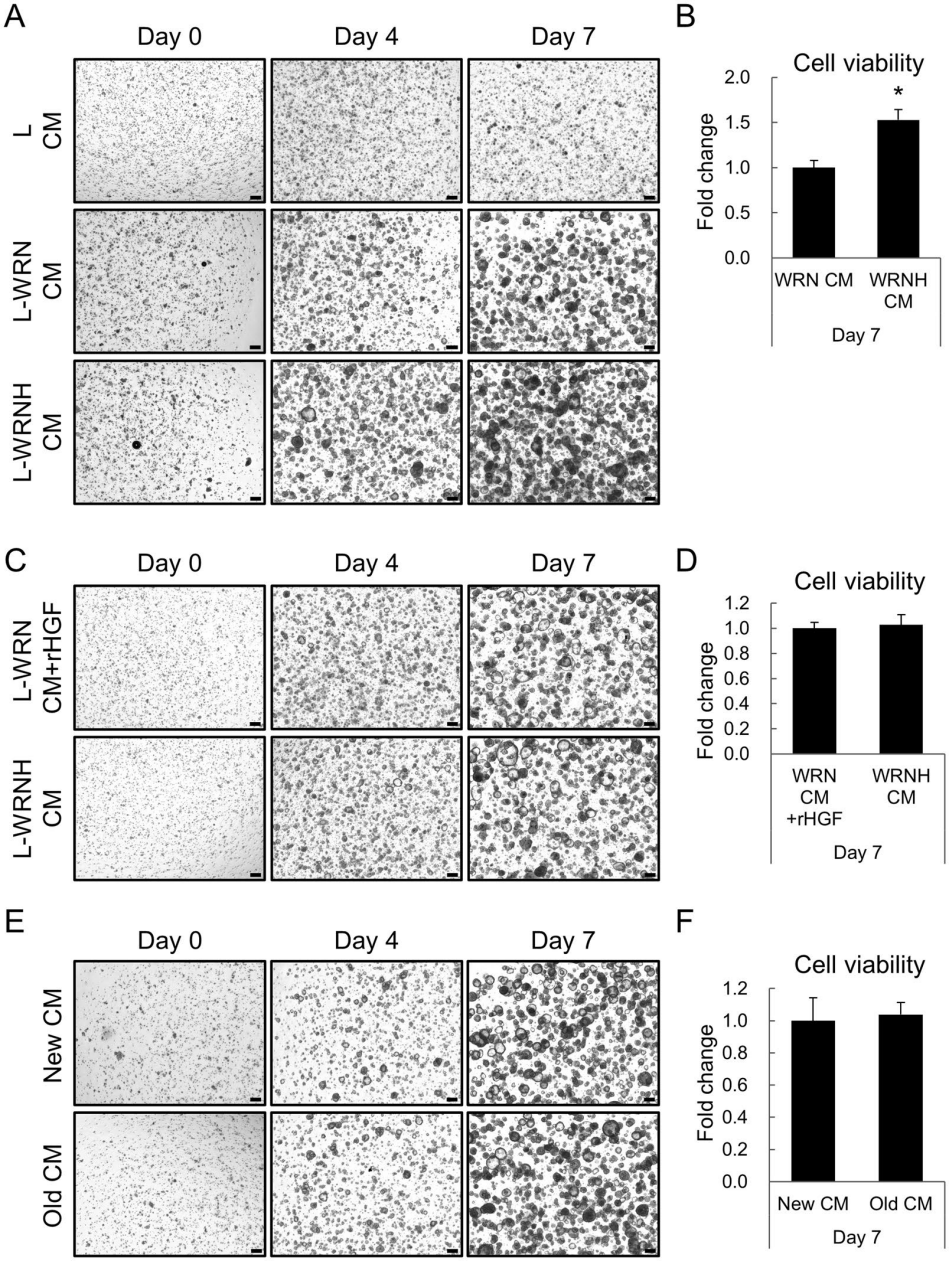
Culture of human intestinal organoids by replacing WRN CM plus recombinant HGF with WRNH CM. (**A–F**) After passage, dispersed hiPSOs embedded in Matrigel were cultured with growth medium containing (**A**, **B**) 25% intact L CM, 25% L-WRN CM, or 25% L-WRNH CM, (**C**, **D**) either 25% L-WRNH CM or 25% L-WRN CM plus 50 ng/mL recombinant HGF, or (**E**, **F**) either 25% L-WRN CM stored at − 80 °C for two years (indicated as "Old CM”) or for two weeks (indicated as “New CM”). (**A**, **C, E**) After 0, 4, and 7 days of culture, the cells were observed with bright field microscopy. A series of z-stack images were then processed to acquire each full-focused image. Scale bar, 200 μm. (**B**, **D, F**) After 7 days of culture, the cells were harvested with TrypLE Express solution and resuspended with basal medium. The number of viable cells was determined using CellTiter-Glo 3D reagents. Assays were performed in n = 4 biologically independent replicates (mean ± S.D.). *P < 0.05.

We cultured hiPSOs with either 25% WRN or 25% WRNH CM and found that hiPSOs cultured with WRNH CM proliferated more prominently than those cultured with WRN CM, as determined by time-dependent microscopic observation (Fig. 1A) and viable cell numbers using CellTiter-Glo 3D reagents (Fig. 1B). Therefore, we compared the 25% WRNH CM with the conventional culture medium (25% WRN CM plus 50 ng/mL recombinant HGF) from the perspective of hiPSO proliferation capacities. The results showed that hiPSOs proliferated similarly in both media through time-dependent microscopic observation (Fig. 1C) and quantification of the number of viable cells after 7 days of passage (Fig. 1D). Notably, CM prepared from intact L cells scarcely proliferated hiPSOs (Fig. 1A), showing that both FBS supplemented in the medium and endogenous secretions from intact L cells had little effect on organoid growth. Importantly, WRNH CM stored at − 80 °C for more than two years did not change the organoid proliferation capacity determined by microscopic images (Fig. 1E) and viable cell numbers (Fig. 1F), indicating that WRNH CM can be stably stored for a long time, which is invaluable in the flexible organoid use. From these data, we conclude that WRNH CM is useful for expanding human intestinal organoids and contributes to their culture cost reduction.

### Matrigel can be replaced with type I collagen gel for human intestinal organoid culture

**Figure 2:**
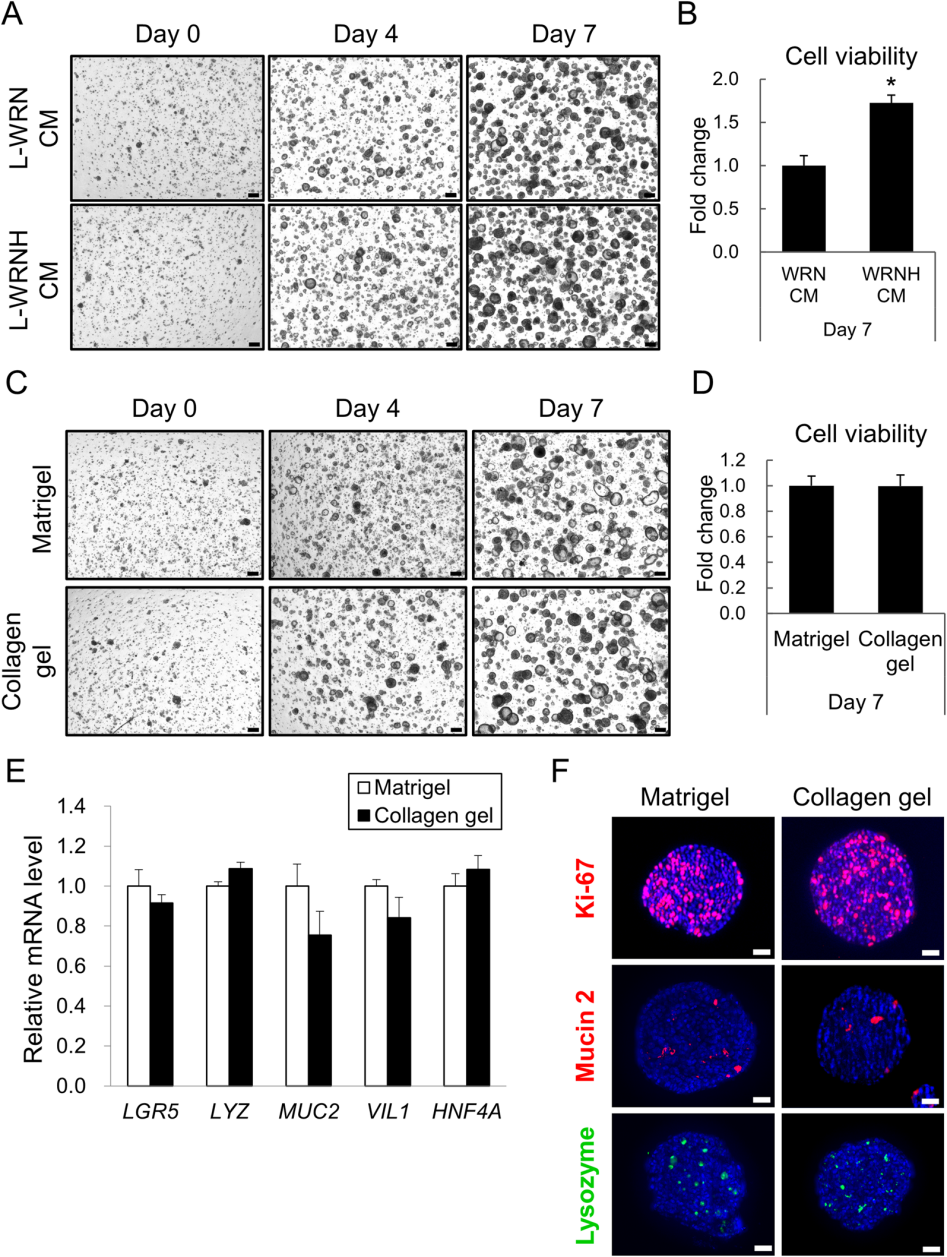
Culture of human intestinal organoids by replacing Matrigel with type I collagen gel. (**A**, **B**) After passage, dispersed hiPSOs embedded in porcine tendon type I collagen gel were cultured with growth medium containing either 25% L-WRN CM or 25% L-WRNH CM. (**C–F**) After passage, dispersed hiPSOs embedded either in Matrigel or porcine tendon type I collagen gel were cultured with the growth medium containing 25% L-WRNH CM. (**A, C**) After 0, 4, and 7 days of culture, the cells were observed with bright field microscopy. A series of z-stack images were then processed to acquire each full-focused image. Scale bar, 200 μm. (**B, D**) After 7 days of culture, the cells were harvested with TrypLE Express solution or 1 mg/mL collagenase solution and resuspended with basal medium. The number of viable cells was determined by CellTiter-Glo 3D reagents. Assays were performed in n = 4 biologically independent replicates (mean ± S.D.). *P < 0.05. (**E**) The cells were harvested after 7 days of the culture, and the mRNA levels of each gene were determined by qRT-PCR and normalized to 18S rRNA levels. Assays were performed in n = 3 biologically independent replicates (mean ± S.D.). (**F**) The cells were harvested after 7 days of the culture, and whole-mount immunostaining was performed using DAPI (blue) together with anti-Ki-67, anti-mucin 2, or anti-lysozyme antibodies. Scale bar, 100 μm.

In addition to changing the culture medium from WRN to WRNH CM, Matrigel was replaced with another ECM to embed organoids for 3D culture. Given that Matrigel is expensive and is derived from mouse sarcoma, which causes lot-to-lot variations and severely limits its use in clinical studies^18^, an alternative candidate should be pure and inexpensive. We selected type I collagen^17^ and alginate hydrogels^19^ as potential ECM for human intestinal organoid culture. Although the alginate hydrogel was much cheaper than the collagen gel, hiPSOs barely proliferated when the gel was used (Figure S1). Type I collagen gel for 3D culture is commercially available, and we chose three more inexpensive products than Matrigel, namely porcine tendon collagen (Nitta Gelatin), bovine dermis collagen (Koken), and bovine dermis atelocollagen (Koken), to compare their suitability for organoid culture. As observed in full-focused z-stack images, hiPSOs embedded in the porcine tendon collagen gel proliferated better than those embedded in the bovine dermis collagen gel (Figure S2). Atelocollagen is a low-immunogenic collagen derivative prepared by removing non-helical telopeptide regions of collagen by protease digestion^20^. Although atelocollagen is somewhat cheaper than collagen, we found that the gel could not maintain stable dome structures during the culture and collapsed as organoids expanded (Figure S2), resulting in the loss of organoids into culture media before harvest. Therefore, we chose the type I collagen derived from the porcine tendon as a potentially suitable matrix for human intestinal organoid culture. As embedded in Matrigel, hiPSOs in the collagen gel proliferated more prominently with WRNH CM than with WRN CM (Fig. 2A, B), emphasizing the effectiveness of WRNH CM in the collagen scaffold. More importantly, time-dependent microscopy observation revealed that hiPSOs in the collagen gel proliferated similarly when using Matrigel (Fig. 2C). Viable cell numbers determined by CellTiter-Glo 3D reagents were also found to be comparable between the two types of gel after 7 days of culture (Fig. 2D). Subsequently, we examined the mRNA levels of various intestinal epithelial genes using qRT-PCR. We found that the expression of the intestinal epithelial stem cell marker gene *LGR5*^21^, Paneth cell marker gene *LYZ*^6^, goblet cell marker gene *MUC2*^22^, mature intestinal epithelial cell marker gene *VIL1*^23^, and intestinal gene that maintains IEC integrity *HNF4A*^24^ was not significantly different between the organoids cultured with Matrigel and the collagen gel (Fig. 2E). Additionally, whole-mount immunostaining revealed that hiPSOs cultured with both Matrigel and the collagen gel contained proliferative cells (Ki67^+^), Paneth cells (lysozyme ^+^), and goblet cells (mucin 2^+^) (Fig. 2F). Furthermore, we also confirmed the continuous growth of hiPSOs in the collagen gel over 10 passages with constant passage dilution rates. These results clearly indicate that the replacement of Matrigel with type I collagen gel did not significantly affect the organoid growth rate or fundamental intestinal marker gene expression, thus ensuring further cost reduction of organoid culture.

### IECs can be developed from hiPSOs and hPIOs cultured with WRNH CM and collagen gel and can be maintained with WRNH CM

**Figure 3:**
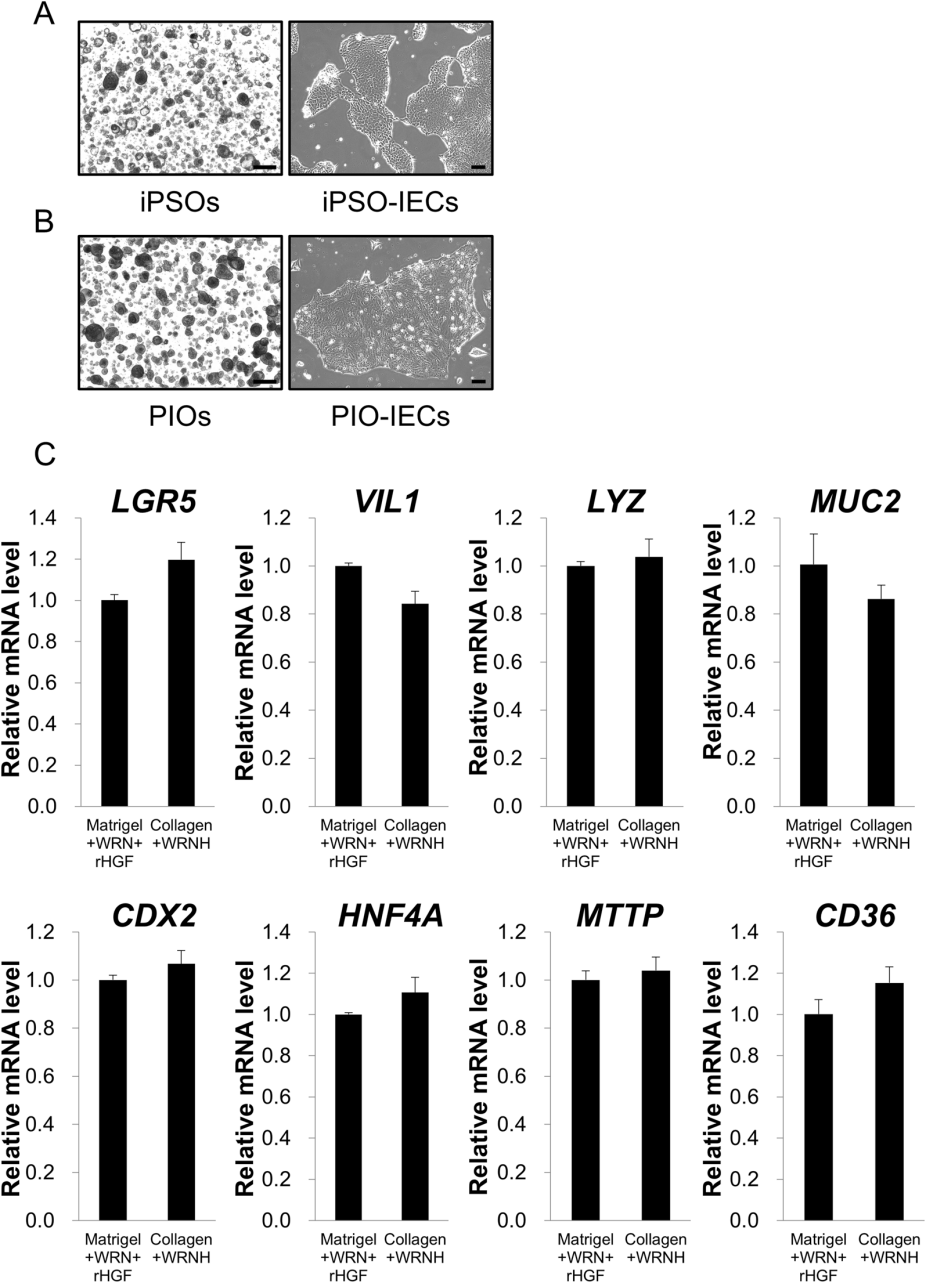
Development of organoid-derived monolayer IECs with WRNH CM and collagen gel. (**A, B**) After passage, hiPSOs (**A**) or hPIOs (**B**) embedded in porcine tendon type I collagen gel were cultured with growth medium containing 25% L-WRNH CM. After 5 days of culture, the cells were observed with bright-field microscopy (*left*). Scale bar, 200 μm. Subsequently, monolayered IECs were developed from either hiPSOs (**A**) or hPIOs (**B**) as described in the Methods, with growth medium containing 25% L-WRN CM, and then observed by phase-contrast microscopy (*right*). Scale bar, 100 μm. (**C**) After passage, hiPSOs embedded either in Matrigel or in porcine tendon type I collagen gel were cultured with growth medium containing 25% L-WRN CM plus 50 ng/mL recombinant HGF or 25% L-WRNH CM, respectively. After 6 days, monolayered IECs were developed from each hiPSOs and were cultured with 25% L-WRN CM plus 50 ng/mL recombinant HGF or 25% L-WRNH CM for 7 days. The cells were then harvested, and the mRNA levels of each gene were determined by qRT-PCR and normalized to 18S rRNA levels. Assays were performed in n = 3 biologically independent replicates (mean ± S.D.).

Despite their usefulness for examining physiological intestinal epithelium function and response, intestinal organoids may not be suitable for studies to evaluate the absorption and/or permeability of nutrients or drug candidates, especially those with high hydrophilicity, since the inside of the organoids corresponds to the intestinal lumen where digested food or xenobiotics physically interact. To overcome this issue, we previously established monolayer IECs from disrupted organoids by trypsinization, which allows exogenous factors to have direct access to the apical (luminal) side of the IECs^10,25^. Since the generation of monolayer IECs at low cost is considered to expand their applications in various assays, we attempted to develop IECs from hiPSOs cultured with WRNH CM and the type I collagen gel before propagating the IECs with WRNH CM. As a result, we confirmed the proliferation of hiPSO-derived IECs (Fig. 3A). Moreover, we previously showed that IECs can also be developed from hPIOs cultured with WRN CM (plus recombinant HGF) and Matrigel^11^. We also confirmed the proliferation of hPIOs with WRNH CM and the collagen gel, as well as the proliferation of hPIO-derived IECs with WRNH CM (Fig. 3B). Furthermore, we examined whether WRNH CM-cultured IECs developed from hiPSOs propagated with collagen gel and WRNH CM exhibited characteristics similar to those of WRN CM plus recombinant HGF-cultured IECs developed from hiPSOs propagated with Matrigel, WRN CM, and recombinant HGF. Gene expression analysis revealed that the mRNA levels of intestinal epithelial marker genes (*LGR5*, *LYZ*, *MUC2, VIL1*, and *CDX2*), as well as intestinal functional genes (*HNF4A*, *MTTP,* and *CD36*), were not statistically different between the IECs (Fig. 3C). These results indicate that physiologically relevant monolayer IECs can be developed from human intestinal organoids using WRNH CM and the type I collagen gel.

### Organoid-derived IEC-selective cytotoxic compounds were identified by high-throughput screening

**Figure 4:**
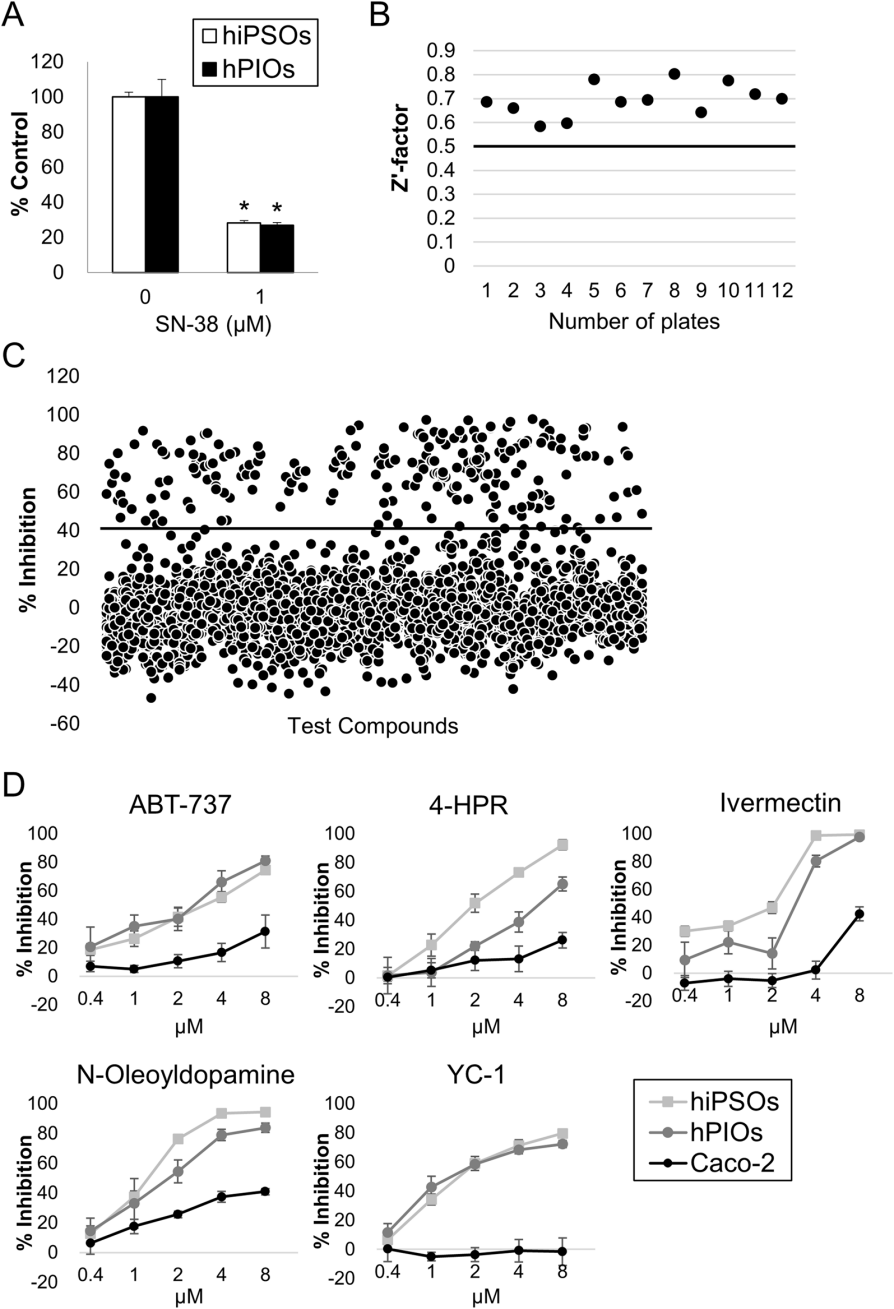
Identification of compounds by compound screening with selective cytotoxicity against organoid-derived IECs. (**A**) Dispersed IECs of hiPSOs and hPIOs were treated with 1 µM SN-38 for 48 h. The number of viable cells was then determined using CellTiter-Glo 3D reagents. Assays were performed in n = 4 biologically independent replicates (mean ± S.D.). *P < 0.05. (**B**) Z′-factor values of the overall primary screening using iPSO-derived IECs. (**C**) Scatterplots of primary screening data at a single concentration of 2 µM in IECs from hiPSOs and selection of hit compounds exhibiting > 40% cytotoxic inhibition activity determined by CellTiter-Glo 3D reagents. (**D**) Dose-dependent analysis of compounds with more selective cytotoxicity against dispersed IECs from hiPSOs and hPIOs than Caco-2 cells. Assays were performed in n = 4 biologically independent replicates (mean ± S.D.).

We recently performed compound screening and discovered that compounds with selective cytotoxicity against Caco-2 cells exclusively include anticancer drugs, highlighting that the origin of Caco-2 cells is cancer cells^11^. To further investigate the different biological responses between intestinal organoids and Caco-2 cells, we performed a large-scale culture of hiPSOs with WRNH CM and the type I collagen gel and conducted another screening of compounds that selectively induce cytotoxicity against hiPSOs from a library of approximately 3500 chemical compounds consisting of drugs and pharmacologically active substances which were provided by Drug Discovery Initiative in the University of Tokyo. Consistent with a previous procedure, the dispersed IECs of organoids were used instead of organoids or monolayered IECs to promote cell homogeneity, which is important for cell-based drug screening to minimize measurement variation^26^. Furthermore, SN-38, a topoisomerase I inhibitor and active metabolite of irinotecan, was chosen as a positive control compound that causes cytotoxicity against IECs because it is known to exhibit intestinal toxicity in vivo^27^. The cytotoxic effect of SN-38 was confirmed by cell viability assay using CellTiter-Glo 3D Reagent, and 1 µM of SN-38 was used to guarantee the assay quality of each plate (Fig. 4A).

The cytotoxic compounds against organoid-derived IECs were first screened at every single concentration of 2 µM in 0.2% dimethyl sulfoxide. The average Z′-factor of the screening exceeded 0.6, indicating that the assay was sufficiently robust to filter the compounds (Fig. 4B). One hundred sixty-seven compounds with > 40% inhibitory activity, which was a statistical cutoff of 4 × standard deviation (SD) of control groups without compound treatment (only with 0.2% dimethyl sulfoxide), were chosen as initial hits (Fig. 4C). Next, the hit compounds were evaluated by a counter-assay to exclude the compounds that are not efficacious against Caco-2 cells. As a result, 62 compounds with < 40% inhibitory activity were selected as potentially selective cytotoxic compounds against organoid-derived IECs (Figure S3). Subsequently, a dose-dependent analysis of these compounds was performed using Caco-2 cells and IECs derived from hiPSOs and hPIOs, and compounds with IEC-selective dose-dependent cytotoxicity were identified.

The identified compounds comprised ABT-737, N-(4-hydroxyphenyl)retinamide (4-HPR), ivermectin, N-oleoyldopamine, and 3-(5′-hydroxymethyl-2′-furyl)-1-benzyl indazole (YC-1) (Fig. 4D). Although these compounds include antiproliferative agents, such as 4-HPR for breast cancer cells^28^, ABT-737 for senescent cells^29^, their effects on normal IECs have not been thoroughly investigated. These results revealed that drug responsiveness is significantly different between normal IECs and Caco-2 cells, which emphasizes the different physiological responses of intestinal organoids and Caco-2 cells.

### YC-1 induces apoptosis through MEK (mitogen-activated protein kinase)/ERK (extracellular signal-regulated kinase) signaling in organoid-derived IEC

**Figure 5:**
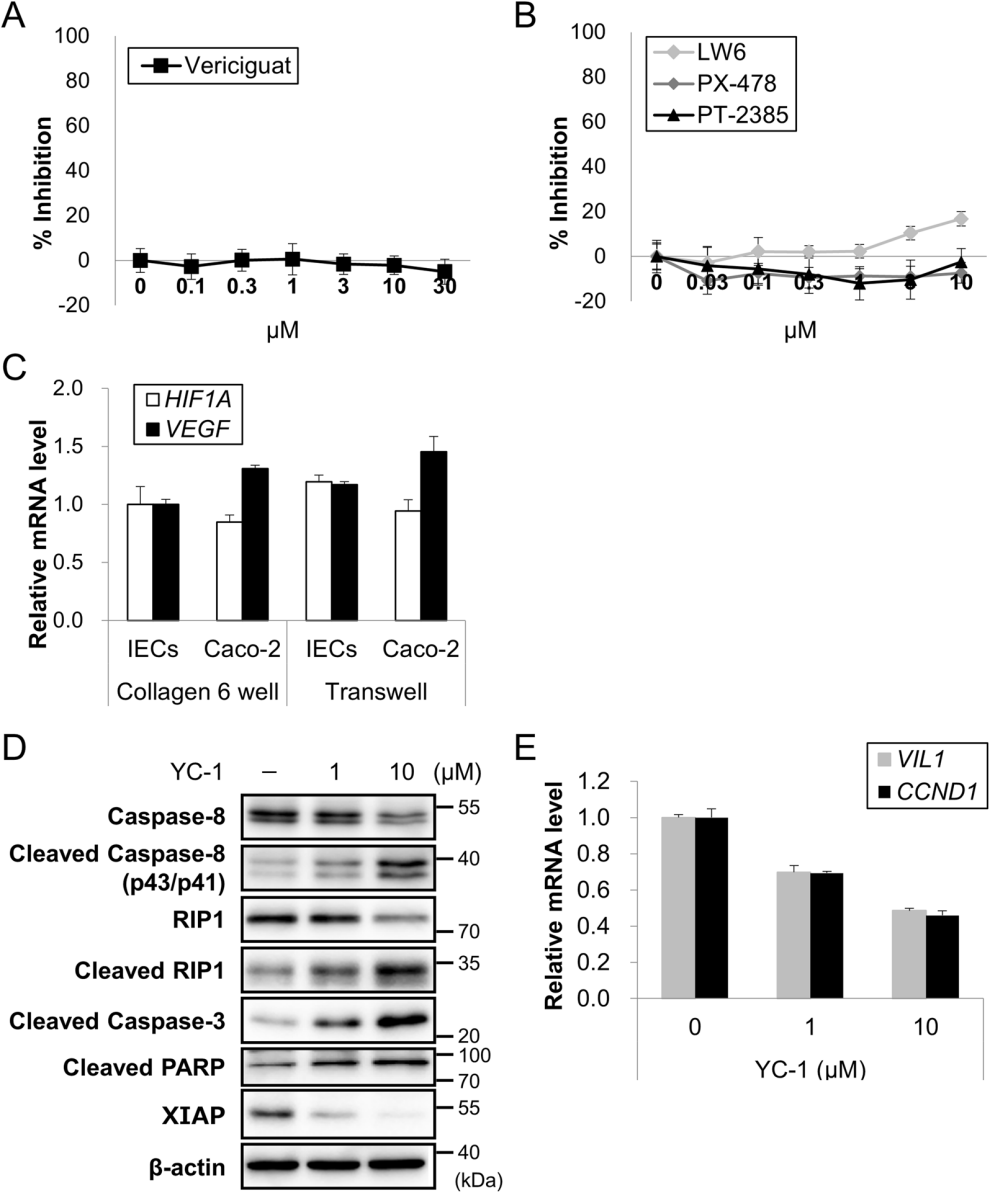
Mechanism of action of YC-1-induced cell death in organoid-derived IECs. (**A, B**) Dispersed hiPSO-derived IECs were treated with vericiguat (**A**) or LW6, PX-478, or PT-2385 (**B**) at the indicated doses for 48 h. Cell viability was determined by CellTiter-Glo 3D reagents. Assays were performed in n = 6 biologically independent replicates (mean ± S.D.). (**C**) Dispersed hiPSO-derived IECs or Caco-2 cells were seeded in collagen I-coated six-well plates or Transwells and cultured as monolayers according to the procedure described in the Methods. After cell harvesting, the *HIF1A* and *VEGFA* mRNA levels were determined by qRT-PCR and normalized to the levels of 18S rRNA. Assays were performed in n = 3 biologically independent replicates (mean ± S.D.). (**D**) Dispersed hiPSO-derived IECs were treated with 0, 1, and 10 µM YC-1 for 14 h. Cells were harvested and western blot analysis was performed using anti-caspase-8, anti-RIP1, anti-cleaved caspase-3, anti-PARP, anti-XIAP, and anti-β-actin antibodies. Full-length blots/gels are presented in Figure S4. (**E**) Dispersed hiPSO-derived IECs were treated with 0, 1, and 10 µM YC-1 for 48 h. The cells were harvested, and the mRNA levels of each gene were determined by qRT-PCR and normalized to 18S rRNA levels. Assays were performed in n = 3 biologically independent replicates (mean ± S.D.).

Among compounds with selective cytotoxicity against organoid-derived IEC, this study focused on YC-1, also called lificiguat, as this compound displayed least cytotoxicity against Caco-2 cells. YC-1 is known as an activator of soluble guanylyl cyclase (sGC) while, at the same time, an inhibitor of hypoxia-inducible factor-1α (HIF-1α)^30,31^. Previously, sGC activation was reported to inhibit human pulmonary arterial smooth muscle cell proliferation^32^ and HIF-1α enhanced the proliferation of certain cells, including mesangial and renal carcinoma cells^33,34^. To elucidate whether the cytotoxic effect of YC-1 on IECs was mediated by sGC activation or HIF-1α inhibition, other chemotypes possessing the same actions as YC-1 namely, vericiguat as an sGC activator and LW6, PX-478, and PT-2385 as HIF-1α inhibitors, were used. Unexpectedly, these compounds cause little toxicity against IECs, suggesting that their cytotoxic effect on IECs was mediated by neither sGC nor HIF-1α (Fig. 5A, B). This conclusion is also supported by data showing that the expression of *HIF1A* and its target gene, *VEGF*, was comparable between monolayered IECs from hiPSOs and Caco-2 cells cultured on collagen I-coated plates or on Transwells where cells represent polarity^10,11^, regardless of their distinct sensitivity to YC-1 (Fig. 5C). Furthermore, we investigated whether YC-1 cytotoxicity was associated with the activation of apoptotic signals. YC-1 dose-dependently induced the cleavage of caspase-8 and its downstream pro-apoptotic proteins, receptor-interacting protein 1 (RIP1), caspase-3, and poly (ADP-ribose) polymerase (PARP), together with decreased the anti-apoptotic protein, X-linked inhibitor of apoptosis (XIAP) (Figs. 5D and S4), indicating the induction of apoptosis. Consistent with a previous finding that YC-1-induced apoptosis accompanies the reduction of *CCND1* expression^33^, the mRNA levels of *CCND1*, as well as the levels of intestinal epithelial cell marker, *VIL1*, were found to be downregulated by the YC-1 treatment (Fig. 5E).

**Figure 6:**
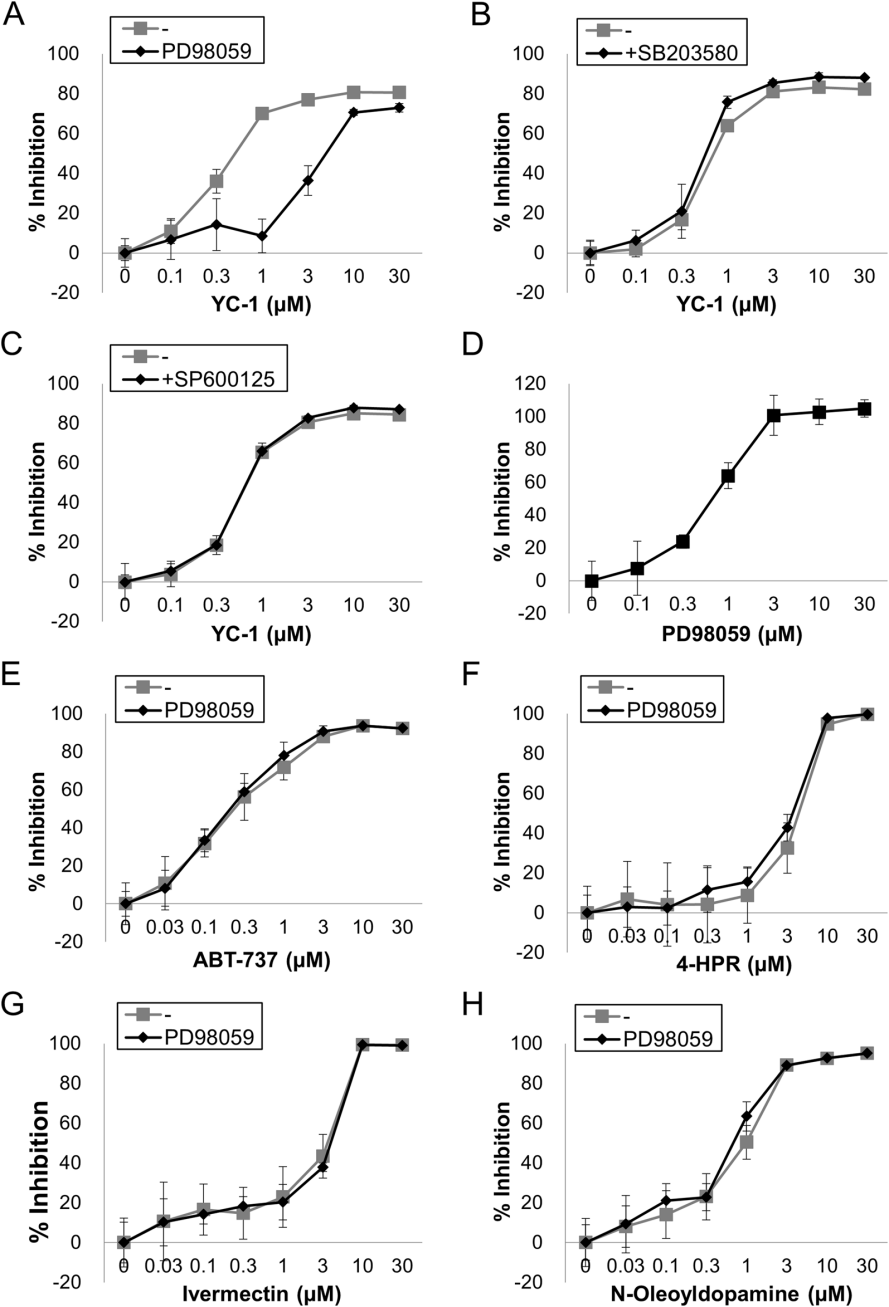
Involvement of MEK/ERK signaling in YC-1-induced apoptosis in organoid-derived IECs. (**A–C**) Dispersed hiPSO-derived IECs were treated with 0, 0.1, 0.3, 1, 3, 10, and 30 µM YC-1 with or without 10 µM PD98059 (**A**), 10 µM SB203580 (**B**), or 10 µM SP600125 (**C**) for 48 h. Cell viability was determined by CellTiter-Glo 3D reagents. Assays were performed in n = 4 biologically independent replicates (mean ± S.D.). (**D**) Dispersed hiPSO-derived IECs were treated with 0, 0.1, 0.3, 1, 3, 10, and 30 µM PD98059 with 1 µM YC-1 for 48 h. Cell viability was determined by CellTiter-Glo 3D reagents. Assays were performed in n = 4 biologically independent replicates (mean ± S.D.). (**E–H**) Dispersed hiPSO-derived IECs were treated with either ABT-737 (**E**), 4-HPR (**F**), ivermectin (**G**), or N-oleoyldopamine (**H**) at concentrations of 0, 0.1, 0.3, 1, 3, 10, and 30 µM with or without 10 µM PD98059 for 48 h. Cell viability was determined by CellTiter-Glo 3D reagents. Assays were performed in n = 4 biologically independent replicates (mean ± S.D.).

In previous studies, YC-1-induced apoptosis was mediated by the activation of the MEK/ERK^35^, p38 mitogen-activated protein kinase (MAPK)^33^, and Jun N-terminal kinase (JNK)^34^ pathways in various cells. To further elucidate the pathway important for apoptosis induction in organoid-derived IECs, inhibitors of each kinase were used. Interestingly, while a p38 MAPK inhibitor (SB203580) and a JNK inhibitor (SP600125) had almost no effect, a MEK/ERK inhibitor (PD98059) substantially prevented the dose-dependent cytotoxicity induced by YC-1 (Fig. 6A–C). Alternatively, PD98059 inhibited the cytotoxicity of 1 µM YC-1 in a dose-dependent manner (Fig. 6D). On the other hand, PD98059 failed to inhibit cytotoxicity caused by ABT-737, 4-HPR, ivermectin, or N-oleoyldopamine (Fig. 6E–H), suggesting a unique molecular mechanism of cytotoxicity induced by YC-1. Finally, PD98059 inhibited YC-1-induced cytotoxicity in IECs from hiPSOs that were expanded with Matrigel (Figure S5), indicating that the difference in ECM composition had little effect on the cellular response to YC-1. Taken together, these results indicate that YC-1 caused cytotoxicity in organoid-derived IECs through the activation of MEK/ERK signaling.

## Discussion

The high physiology of cell models is considered essential for cell-based phenotypic screening, such as drug discovery. Recently, we reported that human organoid-derived IECs can perform crucial intestinal functions that cannot be replicated in Caco-2 cells^11^, suggesting that the use of IECs would enable the evaluation of physiologically relevant in vivo events in a markedly precise manner. To increase their applications, we have tackled the issue caused by conventional organoid culture using commercially available recombinant proteins that incurs high costs and therefore requires cost reduction for routine or large-scale assays. To date, we have succeeded in reducing the cost by using CM harvested from L-WRN cells instead of recombinant proteins. In this study, we showed that further cost reduction could be achieved by replacing WRN CM and recombinant HGF with WRNH CM. Although the generation of WRN CM has been reported to date^36^, which is independent of our lentiviral transduction system, the application of HGF-containing CM to organoid culture has not been reported. HGF is reported to enhance the proliferation of IECs^37^ and can improve intestinal injury in vivo^38,39^, and has been used for human intestinal organoid culture^8,17^. In our organoid culture system, all recombinant proteins were successfully substituted with CM, except for epidermal growth factor (EGF), which is an inexpensive cytokine.

Matrigel is known to be enriched in laminin, which is an effective ECM for organoid cultures^26,40^. However, because laminin is much more expensive than Matrigel, we believe that the use of laminin was not appropriate for the purposes of this study. A four-arm maleimide-terminated poly (ethylene glycol) macromer hydrogel is also a well-defined and appropriate matrix that may replace Matrigel for organoid culture^41^. However, this material is more expensive than type I collagen gel. Collagen gel has been used for the culture of intestinal organoids^42,43^ but much less frequently than Matrigel. However, we demonstrate that human intestinal organoids can be cultured in type I collagen gel at a growth rate similar to that of Matrigel, and monolayer IECs can also be developed from organoids expanded with the collagen gel. To the best of our knowledge, this is the first study to represent the culture of human iPSC-derived normal intestinal organoids in a collagen scaffold. The price of type I collagen gel is approximately one-tenth of that of Matrigel, which can significantly contribute to experimental cost savings. In addition to cost reduction, type I collagen is physiologically relevant as a major ECM in the small intestine^44^, and the use of collagen gel instead of Matrigel will be beneficial, especially for human studies, as it does not contain carcinogenic or unidentified humoral factors that may exert some unexpected effects. Notably, organoid-derived monolayer IECs would facilitate assays, such as absorption of bioactive molecules, intestinal barrier function, and co-culture with other cells, which may be difficult to conduct when using their parental organoids. Therefore, successful IEC culture at low costs can accelerate their use, leading to a more precise understanding of the physiological function of IECs, as well as the development of a new strategy for drug screening and development.

In this study, we proved that hiPSOs can be expanded on a large scale using WRNH CM and collagen type I gel. Through these extensive alterations, the cost associated with human intestinal organoid culture was reduced by up to 100-fold compared to when covering all with conventionally used commercial products (recombinant proteins and Matrigel)^8^. The resulting hiPSOs were used for the screening of thousands of compounds to identify those that selectively induce cellular toxicity against hiPSO-derived IECs. The cytotoxic activity of these compounds was also confirmed when using hPIO-derived IECs. However, due to the potential individual differences, hPIOs derived not from one patient but from many patients may need to be used to estimate the compound toxicity more precisely in the future. Moreover, because the effects of these compounds on intestinal toxicity are currently unknown, there is a need to further investigate the extent to which IEC-selective compounds induce intestinal toxicity in humans. This study focused on YC-1, which exhibited significant cytotoxicity against IECs but very little against Caco-2 cells, and explored its mechanism of action. Considering that YC-1 is one of the HIF-1α inhibitors^31^, and the intestinal tract is a hypoxic and anaerobic environment where HIF-1α can be induced^45^, the toxicity exhibited by YC-1 against IEC was assumed to be associated with HIF-1α. However, other HIF-1α inhibitors with different mechanisms of action have little cytotoxicity effect. Furthermore, among several known pharmacological actions, YC-1 induces MEK/ERK-dependent apoptosis. Although the detailed molecular mechanism is currently unclear, the results demonstrate that intracellular signals are different between Caco-2 cells and normal IECs, suggesting the potential usefulness of IECs in screening studies such as prediction of intestinal toxicity or mechanism of action analysis.

Collectively, we successfully reduced the cost of culturing human intestinal organoids using WRNH CM and type I collagen gel. The method also enabled the culture of monolayered IECs developed from both hiPSOs and hPIOs. We performed compound screening using organoids cultured with WRNH CM and type I collagen gel and identified compounds with higher cytotoxicity against IECs than against Caco-2 cells. These findings provide a basis for organoid research to accelerate a deeper understanding of the physiological relevance of human biology and industrial and clinical applications, including drug screening and transplantation.

## Methods

### Materials

Antibodies against cleaved caspase-3 (9661), caspase-8 (9746), PARP (9542), and RIP1 (3493) were purchased from Cell Signaling Technologies. Antibodies against mucin 2 (sc-7314), and XIAP (sc-55551) were purchased from Santa Cruz Biotechnology. Antibodies against β-actin (A5441), Ki-67 (550609), and lysozyme (A0099) were purchased from Sigma-Aldrich, BD Biosciences, and Dako, respectively. The 4,6-diamidino-2-phenylindole (DAPI) solution was purchased from Cell Biolabs. Secondary antibodies for western blotting and immunostaining were purchased from Jackson ImmunoResearch, except for Alexa Fluor 568 conjugated anti-mouse IgG, which was from Thermo Fisher Scientific. The recombinant mouse EGF, human EGF, human fibroblast growth factor (FGF) 2, and human FGF4 were purchased from PeproTech. The recombinant human/mouse/rat activin A, human R-spondin1, human Noggin, and human HGF were purchased from R&D Systems. A collagen gel culturing kit (638-00781) and type I-A collagen gel (631-00651 or 637-00653) were purchased from Nitta Gelatin (Japan). Collagen gel reconstitution for 3D culture was performed according to the manufacturers’ instruction as follows: 800 μL of Cellmatrix Type I-A and 100 μL of 10 × minimum essential medium (MEM) Culture Solution were mixed by pipetting without bubbling on ice, followed by addition and mixing of 100 μL of Buffer Solution for Reconstitution. Native collagen acidic solution (IAC-50) and atelocollagen acidic solution (IPC-50) were purchased from Koken. Sodium alginate 80-120 was purchased from FUJIFILM Wako Chemicals. Alginate gel formation was conducted as per the previous study^19^.

### Cell culture

Caco-2 cells, obtained from the American Type Culture Collection (HTB-37), were cultured in Eagle’s MEM supplemented with 10% fetal bovine serum (FBS), 1 × MEM non-essential amino acid solution, 100 units/ml penicillin, and 100 µg/mL streptomycin. For differentiation, Caco-2 cells were cultured in a monolayer for > 2 weeks after reaching confluence. The induction of stable transepithelial electrical resistance was confirmed before use in the assays.

L cells stably expressing human R-spondin1, human Noggin, and mouse Wnt3a with (L-WRNH) or without (L-WRN) human HGF were established by lentiviral infection at each suitable dilution ratio, as previously described^8^. The cells were cultured in Dulbecco’s modified Eagle’s medium (DMEM) supplemented with 10% FBS, 100 units/mL penicillin, and 100 µg/mL streptomycin. Each CM was prepared from supernatants seeded in a 1.4 × 10^6^ cells/35 mm plate for 72 h.

The differentiation of human iPS cells (TkDN4-M) into intestinal organoids was performed as previously described^8^. Briefly, subconfluent iPS cells were differentiated into definitive endoderm by treating with Roswell Park Memorial Institute (RPMI) 1640 supplemented with 2 mM L-glutamine, 100 ng/mL activin A, and 20 ng/mL human Wnt3a for 24 h, followed by RPMI 1640 supplemented with 2 mM L-glutamine, 100 ng/mL activin A, 8 ng/mL human FGF2, and 0.2% defined FBS (HyClone) for 24 h, and then RPMI 1640 supplemented with 2 mM L-glutamine, 100 ng/mL activin A, 8 ng/mL human FGF2, and 2% defined FBS for a further 24 h. For mid- and hindgut differentiation, definitive endoderm cells were cultured in RPMI 1640 supplemented with 2 mM L-glutamine, 2% defined FBS, 500 ng/mL human FGF4, and 500 ng/mL human Wnt3a for up to four days. Free-floating spheroids were collected and transferred into three-dimensional cultures in Matrigel refed with Advanced DMEM/F12 supplemented with 15 mM HEPES (pH 7.3), 1 × B-27 (Thermo Fisher Scientific), 1 × N2 (Thermo Fisher Scientific), 2 mM L-glutamine, 100 ng/mL mouse or human EGF, 500 ng/mL human R-spondin1, 100 ng/mL human Noggin, 100 units/mL penicillin, and 100 µg/mL streptomycin, which was replaced every 2–3 days for 14 days.

Human intestinal organoid (hiPSO and hPIO) culture and passage were as follows. Organoids embedded in Matrigel or collagen gel were washed with phosphate-buffered saline (PBS) and treated with either TrypLE Express solution (Thermo Fisher Scientific) for 5 min at 37 °C in a water bath without shaking (Matrigel) or 1 mg/mL collagenase solution for 15 min at 37 °C in a water bath with shaking (collagen gel). The organoids treated with collagenase were then collected by centrifugation at 440×*g* for 3 min, washed twice with 10 ml of basal medium, and suspended in TrypLE Express solution. Subsequently, organoids were disrupted by vigorous pipetting 30–40 times and collected by centrifugation at 440×*g* for 3 min. After removing the supernatant, the organoids were washed with 10 ml of basal medium (Advanced DMEM/F-12 supplemented with 10 mM HEPES (pH 7.3), 2 mM GlutaMAX, 100 units/mL penicillin, and 100 μg/mL streptomycin). After centrifugation at 440×*g* for 3 min, the organoids were resuspended in Matrigel or reconstituted type I collagen gel with 20% growth medium [Advanced DMEM/F-12 with 25% WRNH CM (or 25% WRN CM and 50 ng/mL human HGF), 10 mM HEPES (pH 7.3), 1% bovine serum albumin (BSA), 2 mM GlutaMAX, 50 ng/mL mouse EGF, 10 μM Y-27632, 10 μM SB202190, 500 nM A83-01, 100 μg/mL gentamicin, 100 units/mL penicillin, and 100 μg/mL streptomycin] on ice. Suspensions were aliquoted into the wells of Nunc 4-well plates (Thermo Fisher Scientific), ensuring that the border of each well was untouched, and solidified in a 5% CO_2_ incubator at 37 °C for 15 min (Matrigel) or 30 min (collagen gel). Growth medium (500 µL) was then added to each well. The entire medium was changed every 3 days. Organoid passage was performed every 6–7 days. The passaging ratios ranged from 1:8 to 1:16. All the cultures were incubated in a 5% CO_2_ incubator at 37 °C. The experiments using primary human ileum organoids, which were established from an 82-year-old Japanese female surgical specimen, complied with the Declaration of Helsinki and were approved by the human ethical committee of The University of Tokyo (No. 18-341) and Osaka University (No. 27-5-11). The tissue was sampled with informed consent. No other experiments on humans were conducted.

### Monolayer culture of organoid-derived cells

After harvesting from the Matrigel or collagen gel, digestion, and disruption by pipetting 30 times in TrypLE Express solution, organoids were collected by centrifugation at 440×*g* for 3 min. After washing with basal medium and harvesting by centrifugation, the cells were resuspended with growth medium, filtered through a 40-μm nylon mesh (Corning), and seeded in six-well plates coated with type I collagen or Transwells (all from Corning). The medium was replaced every 2–3 days. Cells were cultured in a 5% CO_2_ incubator at 37 °C.

### Quantitative reverse transcription-polymerase chain reaction (qRT-PCR)

Total cellular RNA was extracted using RNeasy Mini Kit (Qiagen). Reverse transcription was performed using a high-capacity cDNA Reverse Transcription Kit (Thermo Fisher Scientific). The mRNA levels were measured by fluorescence real-time PCR on StepOnePlus (Thermo Fisher Scientific) using PrimeTime qPCR Probes (Integrated DNA Technologies). The 18S rRNA levels were used as an internal control to normalize the mRNA levels of each gene.

### Western blot analysis

After the preparation of lysis buffer [50 mM Tris-HCl (pH 8.0), 150 mM NaCl, 1% Triton X-100, 0.5% deoxycholate, 0.1% sodium dodecyl sulfate (SDS), and a protease inhibitor cocktail (Nacalai Tesque)], cell lysates were subjected to SDS-polyacrylamide gel electrophoresis and transferred onto a polyvinylidene difluoride membrane. The membrane was blocked with Blocking One (Nacalai Tesque) for 1 h at room temperature (RT), incubated overnight at 4 °C with primary antibodies against caspase-3 (1:1000), caspase-8 (1:1000), PARP (1:1000), RIP1 (1:1000), XIAP (1:100), and β-actin (1:200), and incubated with secondary antibodies for 1 h at RT. Chemiluminescent signals were determined using Fusion Solo S (Vilber). A pre-stained ladder protein marker (XL-Ladder Broad, APRO Science) that was not visible as a chemiluminescent signal was used to determine the position of molecular weights. Full-scan images were represented in the Supplementary Information.

### Immunofluorescence staining

Whole-mount immunofluorescence staining of organoids was performed according to a previous protocol^8^. Briefly, organoids were fixed and permeabilized using a Cytofix/Cytoperm Kit (BD Biosciences). The specimens were then incubated with primary antibodies [rabbit anti-lysozyme (1:10), mouse anti-mucin 2 (1:50), or mouse anti-Ki67 (1:50)] at 4 °C overnight. After washing three times with Perm/Wash Buffer, organoid specimens were further incubated with Alexa Fluor 568-conjugated anti-mouse IgG (1:800) or Alexa Fluor 647-conjugated anti-mouse IgG (1:100) for 3 h at 4 °C. After washing three times with Perm/Wash Buffer, the cells were incubated with DAPI (1:1000) for 10 min at RT. Fluorescence staining was visualized with fully focused z-stack images using an all-in-one fluorescence microscope (Keyence, Japan) equipped with structured illumination.

### Determination of cell viability

After harvesting from the Matrigel and disrupting by pipetting 30 times in TrypLE Express solution, organoids were collected by centrifugation at 440×*g* for 3 min. After washing with basal medium and harvesting by centrifugation, the cells were resuspended in growth medium and filtered through a 40-μm nylon mesh, resulting in a dispersed state at the single-cell level. The cells were then seeded at 5.0 × 10^3^ per well (96-well plate) or 1.2 × 10^3^ per well (384-well plate) with or without the compounds and incubated for 48 h in a 5% CO_2_ incubator at 37 °C. After CellTiter-Glo® 3D reagent (Promega) was added to each well and incubated for 10 min at RT, luminescence signals were measured using TriStar2 (Berthold) or PHERAstar (BMG Labtech), according to the manufacturers’ protocols.

### Statistics

Results are presented as the mean ± standard deviation (SD). Data were analyzed using Student's t-test for two groups. Differences were considered significant at *P* < 0.05 (indicated by asterisks).

## Supplementary Information

Supplementary Information.

## Data Availability

The datasets used during the current study are available from the corresponding author upon reasonable request.

## Abbreviations

BSA: Bovine serum albumin
CM: Conditioned medium
DAPI: 4,6-Diamidino-2-phenylindole
DMEM: Dulbecco’s modified eagle’s medium
ECM: Extracellular matrix
EGF: Epidermal growth factor
ERK: Extracellular signal-regulated kinase
FBS: Fetal bovine serum
FGF: Fibroblast growth factor
HGF: Hepatocyte growth factor
HIF-1α: Hypoxia-inducible factor-1α
hiPSO: Human iPS cell-derived intestinal organoids
hPIO: Primary human ileum organoids
4-HPR: N-(4-Hydroxyphenyl)retinamide
IECs: Intestinal epithelial cells
iPS: Induced-pluripotent stem
JNK: Jun N-terminal kinase
MAPK: Mitogen-activated protein kinase
MEK: Mitogen-activated protein kinase
MEM: Minimum essential medium
PARP: Poly (ADP-ribose) polymerase
PBS: Phosphate-buffered saline
RIP1: Receptor-interacting protein 1
RPMI: Roswell Park Memorial Institute
RT: Room temperature
qRT-PCR: Quantitative reverse transcription-polymerase chain reaction
SD: Standard deviation
SDS: Sodium dodecyl sulfate
sGC: Soluble guanylyl cyclase
WRN: Wnt3a, R-spondin1, and Noggin
WRNH: Wnt3a, R-spondin1, Noggin, and HGF
XIAP: X-linked inhibitor of apoptosis
YC-1: 3-(5′-Hydroxymethyl-2′-furyl)-1-benzyl indazole
