# Supplementary material for: Drug cytotoxicity screening using human intestinal organoids propagated with extensive cost-reduction strategies
Source: Sci Rep. 2023 Apr 3;13:5407. doi: 10.1038/s41598-023-32438-2 (PMC10070462; doi:10.1038/s41598-023-32438-2)
Supplement: Supplementary file 1 — Supplementary Information. [file 41598_2023_32438_MOESM1_ESM.pdf]

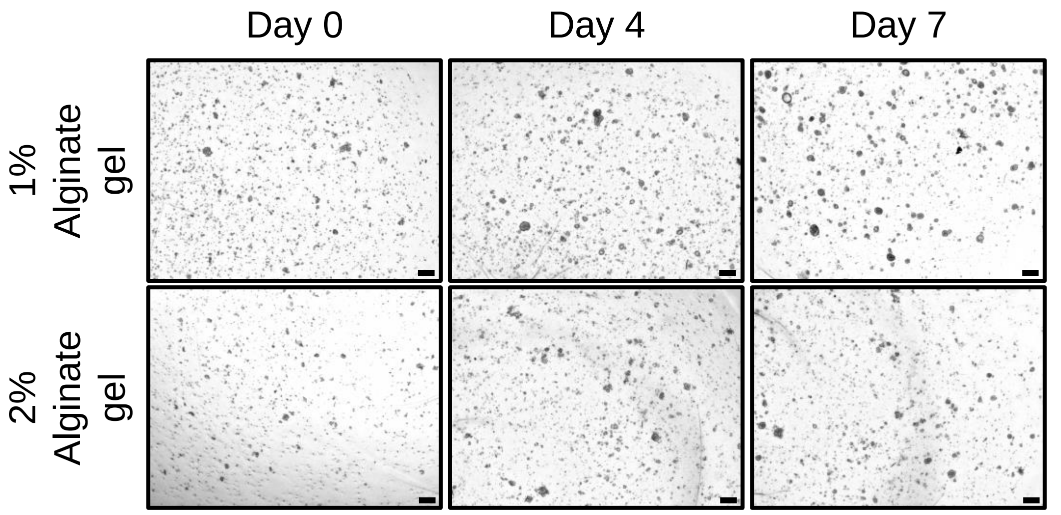

Figure S1. Little proliferative activity of human intestinal organoids in alginate gel. After passage, dispersed hiPSOs embedded in 1 or 2% alginate gel were cultured with growth medium containing 25% L-WRNH CM. After 0, 4, and 7 days of culture, the cells were observed with bright field microscopy. A series of z-stack images were then processed to acquire each full-focused image. Scale bar, 200 μm.

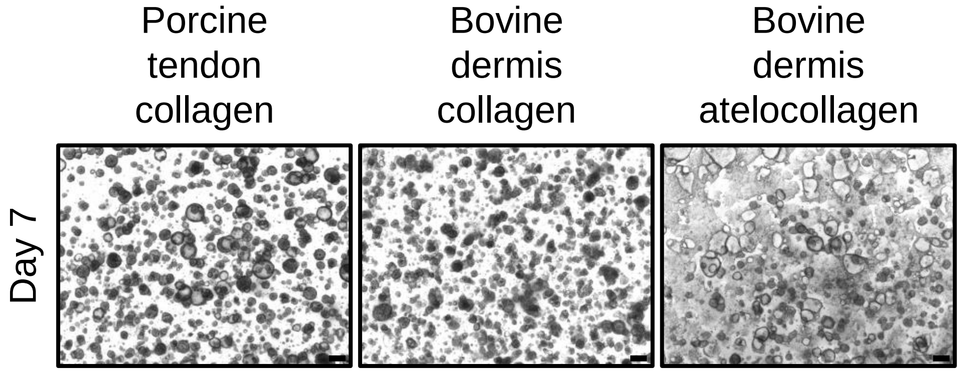

Figure S2. Comparison of proliferation and morphology of human intestinal organoids among three types of commercially available type I collagen gel. After passage, dispersed hiPSOs embedded in porcine tendon collagen gel, bovine dermis collagen gel, or bovine dermis atelocollagen gel were cultured with growth medium containing 25% L-WRNH CM. After 7 days of culture, the cells were observed with bright field microscopy. A series of z-stack images were then processed to acquire each full-focused image. Scale bar, 200 μm.

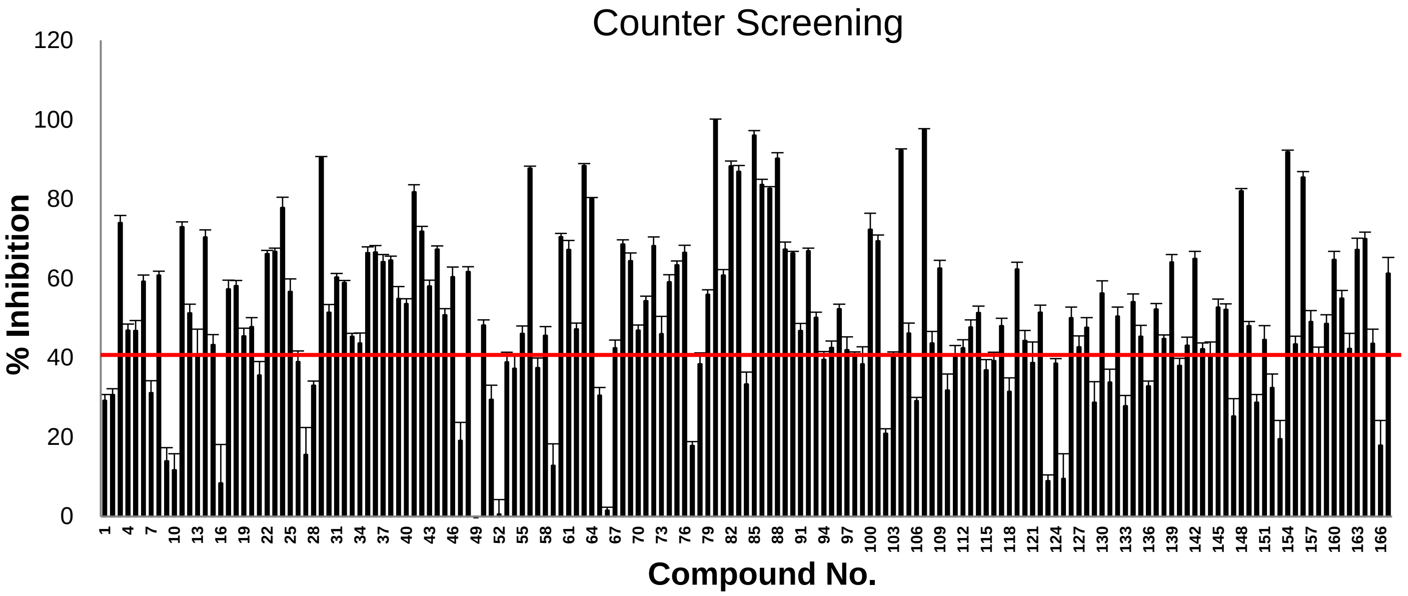

Figure S3. Screening of compounds with potentially less cytotoxicity in Caco-2 cells. Caco-2 cells suspended with culture medium were seeded at 1.2×10^3 per well (384-well plate) and treated with 167 compounds selected by first screening using hiPSOs at a concentration of 2 µM, and compounds were selected exhibiting <40% cytotoxic inhibition activity determined by CellTiter-Glo 3D reagents. Assays were performed in n = 4 biologically independent replicates (mean ± S.D.).

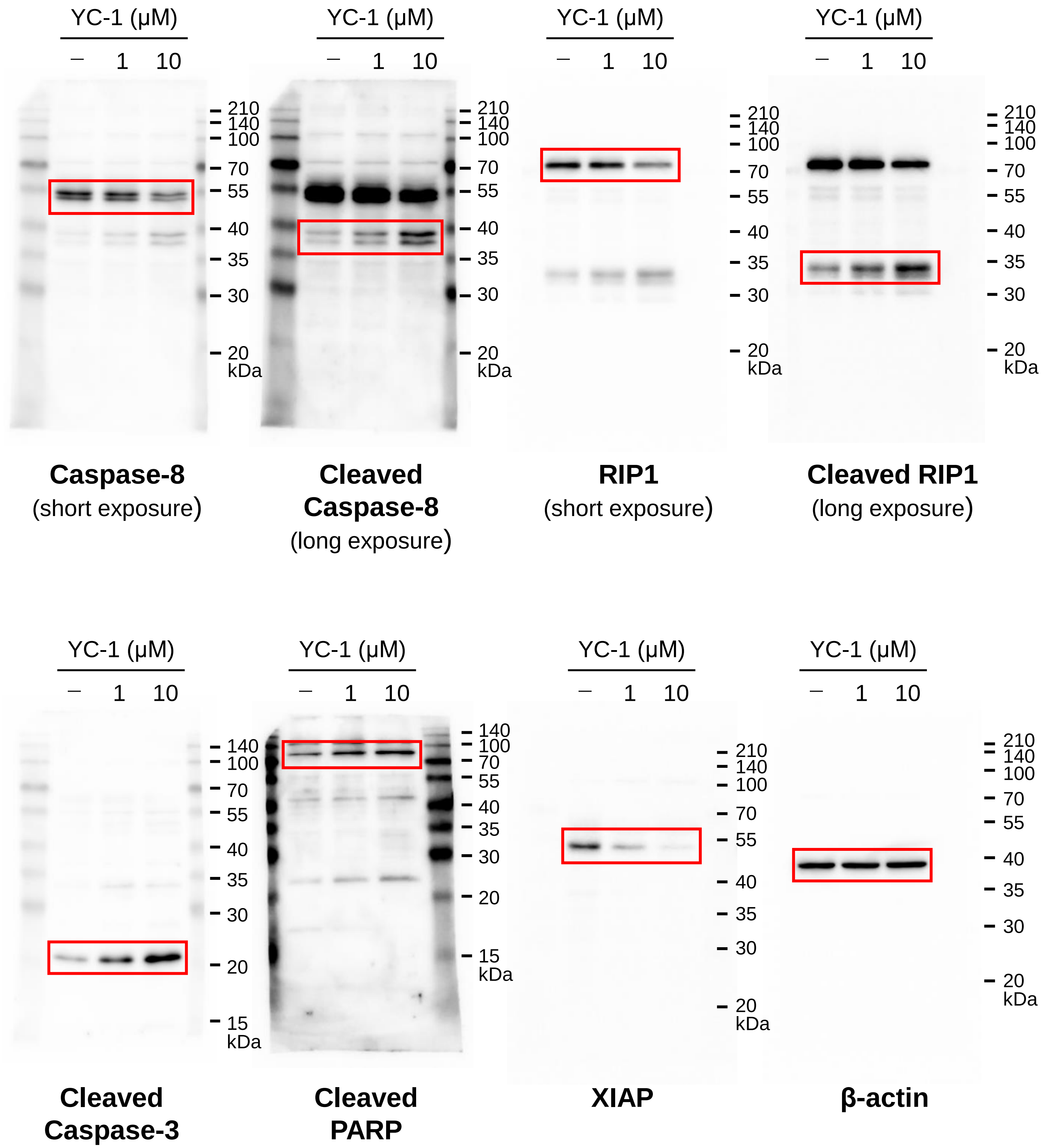

Figure S4. Full-scan images of Western blot analysis in Figure 5D. Red squares indicate the bands represented in Figure 5D. A pre-stained ladder protein marker that was not visible as a chemiluminescent signal was used to determine the position of molecular weights.

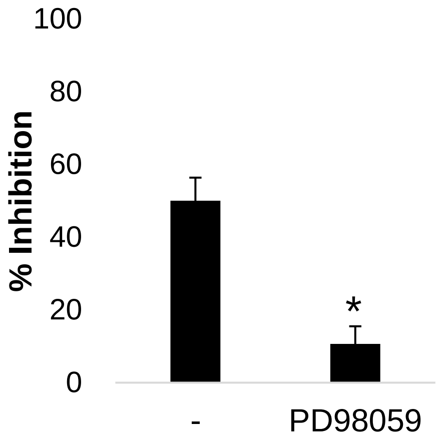

Figure S5. Inhibition of YC-1-induced apoptosis by PD98059 in IECs developed from organoids cultured with Matrigel. Dispersed IECs from hiPSOs cultured in Matrigel were treated with 1 µM YC-1 with or without 10 µM PD98059 for 48 h. Cell viability was determined by CellTiter-Glo 3D reagents. Assays were performed in n = 4 biologically independent replicates (mean ± S.D.) $*P < 0.05$.
